# Supplementary material for: Modeling glioblastoma heterogeneity as a dynamic network of cell states
Source: Mol Syst Biol. 2021 Sep 16;17(9):e10105. doi: 10.15252/msb.202010105 (PMC8444284; doi:10.15252/msb.202010105)
Supplement: Supplementary file 5 — Source Data for Figure 3 [file MSB-17-e10105-s001.zip › Figure3A_sourcedata/GSEA_3065/nosigdb_state6.GseaPreranked.1622718443993/OLSEN_ADRENERGIC.html]

Details for gene set OLSEN\_ADRENERGIC[GSEA]

|  || Dataset | state6 |
| Phenotype | NoPhenotypeAvailable |
| Upregulated in class | na\_pos |
| GeneSet | OLSEN\_ADRENERGIC |
| Enrichment Score (ES) | 0.51994646 |
| Normalized Enrichment Score (NES) | 2.0081728 |
| Nominal p-value | 0.0 |
| FDR q-value | 0.001270616 |
| FWER p-Value | 0.011 |
Table: GSEA Results Summary

  

Fig 1: Enrichment plot: OLSEN\_ADRENERGIC      
 Profile of the Running ES Score & Positions of GeneSet Members on the Rank Ordered List

  

| PROBE | GENE SYMBOL | GENE\_TITLE | RANK IN GENE LIST | RANK METRIC SCORE | RUNNING ES | CORE ENRICHMENT || 1 | SOX4 |  |  | 3 | 0.375 | 0.0563 | Yes |
| 2 | SOX11 |  |  | 70 | 0.230 | 0.0836 | Yes |
| 3 | PTMA |  |  | 94 | 0.216 | 0.1136 | Yes |
| 4 | H3F3A |  |  | 104 | 0.212 | 0.1446 | Yes |
| 5 | RPS7 |  |  | 107 | 0.210 | 0.1761 | Yes |
| 6 | HNRNPA1 |  |  | 108 | 0.209 | 0.2076 | Yes |
| 7 | MZT2B |  |  | 113 | 0.206 | 0.2383 | Yes |
| 8 | RPL38 |  |  | 182 | 0.182 | 0.2580 | Yes |
| 9 | TMSB15A |  |  | 253 | 0.161 | 0.2744 | Yes |
| 10 | HINT1 |  |  | 274 | 0.156 | 0.2957 | Yes |
| 11 | CRMP1 |  |  | 314 | 0.149 | 0.3137 | Yes |
| 12 | GAP43 |  |  | 315 | 0.149 | 0.3362 | Yes |
| 13 | RPL27 |  |  | 382 | 0.139 | 0.3496 | Yes |
| 14 | KIF5C |  |  | 415 | 0.134 | 0.3661 | Yes |
| 15 | YWHAQ |  |  | 478 | 0.125 | 0.3780 | Yes |
| 16 | H3F3B |  |  | 514 | 0.122 | 0.3924 | Yes |
| 17 | PAFAH1B3 |  |  | 536 | 0.120 | 0.4080 | Yes |
| 18 | ZNF428 |  |  | 553 | 0.118 | 0.4239 | Yes |
| 19 | CD24 |  |  | 674 | 0.108 | 0.4265 | Yes |
| 20 | ATP6V0E2 |  |  | 676 | 0.108 | 0.4427 | Yes |
| 21 | HMGN2 |  |  | 679 | 0.108 | 0.4587 | Yes |
| 22 | SUMO2 |  |  | 710 | 0.105 | 0.4711 | Yes |
| 23 | RBP1 |  |  | 788 | 0.099 | 0.4773 | Yes |
| 24 | TUBB2B |  |  | 795 | 0.099 | 0.4915 | Yes |
| 25 | CHCHD2 |  |  | 796 | 0.099 | 0.5064 | Yes |
| 26 | CDK6 |  |  | 807 | 0.097 | 0.5199 | Yes |
| 27 | CCND1 |  |  | 1317 | 0.071 | 0.4723 | No |
| 28 | BEX1 |  |  | 1352 | 0.069 | 0.4789 | No |
| 29 | CKB |  |  | 1359 | 0.069 | 0.4886 | No |
| 30 | FSCN1 |  |  | 1498 | 0.064 | 0.4824 | No |
| 31 | STMN1 |  |  | 1649 | 0.058 | 0.4741 | No |
| 32 | OCIAD2 |  |  | 2061 | 0.046 | 0.4340 | No |
| 33 | JPT1 |  |  | 2094 | 0.045 | 0.4371 | No |
| 34 | TUBA1A |  |  | 2135 | 0.044 | 0.4391 | No |
| 35 | SLC38A1 |  |  | 2301 | 0.039 | 0.4260 | No |
| 36 | MLLT11 |  |  | 2335 | 0.038 | 0.4279 | No |
| 37 | CCT3 |  |  | 2419 | 0.036 | 0.4238 | No |
| 38 | CBX1 |  |  | 2863 | 0.025 | 0.3768 | No |
| 39 | MAP1B |  |  | 2893 | 0.024 | 0.3772 | No |
| 40 | MAPT |  |  | 3232 | 0.017 | 0.3410 | No |
| 41 | NREP |  |  | 3707 | 0.007 | 0.2877 | No |
| 42 | PPIA |  |  | 3964 | 0.001 | 0.2586 | No |
| 43 | RTN1 |  |  | 4676 | -0.012 | 0.1790 | No |
| 44 | CALM2 |  |  | 5062 | -0.020 | 0.1379 | No |
| 45 | HDAC2 |  |  | 5329 | -0.025 | 0.1112 | No |
| 46 | HAND2 |  |  | 5361 | -0.026 | 0.1115 | No |
| 47 | TAGLN3 |  |  | 5582 | -0.030 | 0.0909 | No |
| 48 | ENO2 |  |  | 5889 | -0.036 | 0.0612 | No |
| 49 | H2AFZ |  |  | 6079 | -0.040 | 0.0456 | No |
| 50 | NCAM1 |  |  | 6342 | -0.046 | 0.0225 | No |
| 51 | ZFHX3 |  |  | 6506 | -0.050 | 0.0113 | No |
| 52 | HSP90AB1 |  |  | 6619 | -0.053 | 0.0066 | No |
| 53 | TUBB |  |  | 6820 | -0.059 | -0.0074 | No |
| 54 | TUBB2A |  |  | 7078 | -0.067 | -0.0267 | No |
| 55 | COX6A1 |  |  | 7089 | -0.067 | -0.0177 | No |
| 56 | RAN |  |  | 7221 | -0.072 | -0.0219 | No |
| 57 | KIF21A |  |  | 7417 | -0.078 | -0.0324 | No |
| 58 | HIST1H4C |  |  | 7641 | -0.088 | -0.0447 | No |
| 59 | UCHL1 |  |  | 7848 | -0.098 | -0.0534 | No |
| 60 | HSP90AA1 |  |  | 8153 | -0.117 | -0.0705 | No |
| 61 | HMGB2 |  |  | 8388 | -0.145 | -0.0754 | No |
| 62 | FABP5 |  |  | 8414 | -0.148 | -0.0559 | No |
| 63 | LUC7L3 |  |  | 8631 | -0.203 | -0.0500 | No |
| 64 | SCG2 |  |  | 8793 | -0.455 | 0.0003 | No |
Table: GSEA details [plain text format]

  

Fig 2: OLSEN\_ADRENERGIC: Random ES distribution      
 Gene set null distribution of ES for **OLSEN\_ADRENERGIC**
